# Advancements and challenges in blood pressure monitoring using pulse wave propagation: a comprehensive review and ISO 81060-2 based statistical analysis

**DOI:** 10.1038/s41440-026-02651-3

**Published:** 2026-05-07

**Authors:** Yang Yu, Andrew Lowe

**Affiliations:** AUT Institute of Biomedical Technologies (IBTec), Auckland University of Technology, Auckland, New Zealand

**Keywords:** Cuffless blood pressure monitoring, Pulse wave propagation, Device accuracy, Digital hypertension, Implemental hypertension, Morning hypertension

## Abstract

Cardiovascular diseases, particularly hypertension, remain a major global health burden, highlighting the need for accurate and accessible blood pressure (BP) monitoring. Cuffless BP measurement (BPM) based on pulse wave propagation methods (PWPM), including pulse arrival time (PAT), pulse transit time (PTT), and pulse wave velocity (PWV), has attracted increasing research interest. This review comprises two components. First, a narrative review of studies published up to June 2025 examines sensing technologies, mathematical models, and validation protocols used in PWPM-based BPM. Second, a statistical re-evaluation of 22 studies published between 2015 and 2025 was conducted using the Credence of Device Acceptability (CDA) and the Probability of Tolerable Error (PTE), grounded in the statistical principles of ISO 81060-2. Accuracy varied widely across physiological conditions, sensing technologies, and study designs, with no single approach demonstrating consistent superiority. The re-evaluation provided a more stringent assessment of performance: only five studies achieved CDA values exceeding 0.95 for both systolic and diastolic BP. Overall, diastolic BP estimation demonstrated superior accuracy compared with systolic BP. Incorporating physiological indices such as arterial compliance and sympathetic activity may improve the robustness and accuracy of BP estimation models. While machine learning shows promise for enhanced feature extraction, calibration tolerance and real-world reliability remain critical challenges. Importantly, the evaluation and development of cuffless BPM technologies should align with validation standards appropriate to the intended application. We recommend that future early-stage studies apply the CDA and PTE framework as supportive accuracy metrics to better assess methodological performance and inform device development and validation.

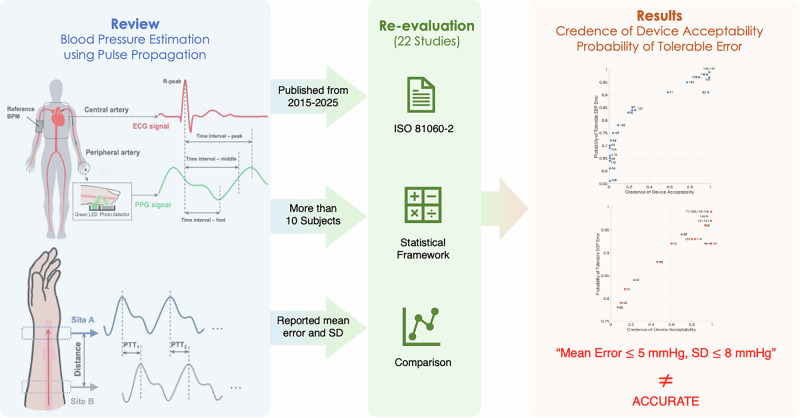

## Introduction

### The importance of blood pressure measurement

The morbidity and mortality of cardiovascular disease are the highest in the current disease spectrum worldwide. According to the World Health Organization’s 2023 report, approximately 1.28 billion adults aged 30 to 79 are living with hypertension globally, and this number is projected to rise to 1.5 billion by 2025. Alarmingly, fewer than 20% of those affected have their blood pressure under control [1]. Hypertension management is always a formidable task, from accurate measurement to effective treatment [2]. The American Heart Association classifies blood pressure (BP) and corresponding diagnosis in four categories according to the systolic blood pressure (SBP) and diastolic blood pressure (DBP) levels: normal, elevated, stage 1 hypertension, and stage 2 hypertension [3]. The blood pressure monitor/measurement (BPM) is a well-known clinical method to monitor cardiovascular function, and it is also a strong predictor of death and cardiovascular disease [4].

**Fig. 1:**
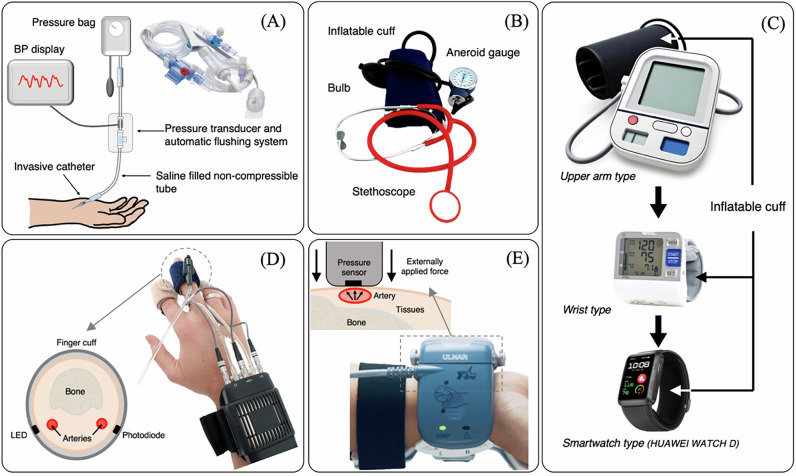
Conventional BPM methods: **A** invasive cannulation method; **B** conventional manual sphygmomanometer using auscultation method; **C** automatic sphygmomanometers using oscillometry method; **D** Finapres® using volume-clamp method; **E** Tonometry using vascular unloading method

Figure 1 visually illustrates the main methods for conventional BPM. The invasive (intra-arterial) cannulation method, regarded as the gold standard for beat-to-beat BP measurement by directly sensing BP via a catheter and transducer [5]. Sphygmomanometer is a classic cuff-based BPM based on principles of auscultation [6] or oscillometry [7]. Auscultation is also used as the reference (ground truth) method according to the international standard ISO 81060-2:2019 [8].

Finapres® NOVA technology [9] is an automated system for continuous BPM using volume clamp principles at the fingers [10]. Compared with the auscultation device, the agreement was especially good for SBP but not DBP [11, 12]. Tonometry (vascular unloading method) is another non-invasive continuous method by applying a probe flattens or applanates the artery to press it against the bone [13], while its accuracy is inconsistent between different research [14]. Belani et al. described a non-invasive device Vasotrac (Medwave, Arden Hills, MN) [15, 16]. Its working principle is to detect the zero-load state of the radial artery at the wrist by generating compression and decompression frequently.

Over the years, numerous leading organizations have developed clinical validation protocols to ensure the accuracy and reliability of BP devices, such as the American National Standards Institute (ANSI)/ the Association for the Advancement of Medical Instrumentation (AAMI) [8], the BHS Protocol by the British Hypertension Society [17], and the ESH International Protocol by the European Society of Hypertension [18]. International regulations have converged on the current ISO 81060-2:2019. With the emergence of cuffless and wearable BPM technologies, which fall outside the scope of ISO 81060-2, standards bodies have responded by introducing new frameworks, including the IEEE 1708a-2019 standard for Wearable, Cuffless Blood Pressure Measuring Devices [19], ISO 81060-3:2022 Non-invasive sphygmomanometers Part 3: Clinical investigation of continuous automated measurement type [20]. The ESH has recently issued comprehensive recommendations specifically addressing the validation of cuffless devices, recognizing that these technologies differ fundamentally from conventional cuff-based monitors in measurement principles, calibration requirements, output frequency, and intended clinical use [21]. Furthermore, a dedicated international standard ISO 81060-7 for intermittent or repeated intermittent cuffless BPM devices is under development. These standards offer harmonized procedures across all stages of clinical evaluation, from validation design to statistical analysis [22] and their adoption is critical for ensuring that emerging BPM technologies demonstrate acceptable performance characteristics for their intended use in real-world applications.

By 2025, the global market for BPM devices is expected to exceed USD 2 billion, growing annually at 9.1% [23]. Wrist monitors are anticipated to become more popular due to their ability to provide multi-parameter readings and integrate with smartphones [24]. Healthcare practitioners commonly employ automated devices for office or clinic-based and 24-h ambulatory BPM [25]. The brachial cuff-based device is a widely-used BPM at home s, but it was reported that over 30% of them are inaccurate [26]. Cuff-based BPM is likely to underestimate SBP and overestimate DBP compared with the invasive method [27]. If it is unaware, an incorrect diagnosis or wrong treatment decision might be made [28].

Unlike traditional cuff-based devices, cuffless BPM aims to provide continuous measurements, offering new opportunities for BP monitoring and management. However, their classification, reliability, and clinical use remain uncertain [29]. Guidelines advise against using cuffless devices for diagnosis or treatment until validated against appropriate standards [30]. For consumers, the accreditation and ratings for BPM devices are available online by Medaval [31].

### Blood pressure estimation using pulse wave propagation

**Fig. 2:**
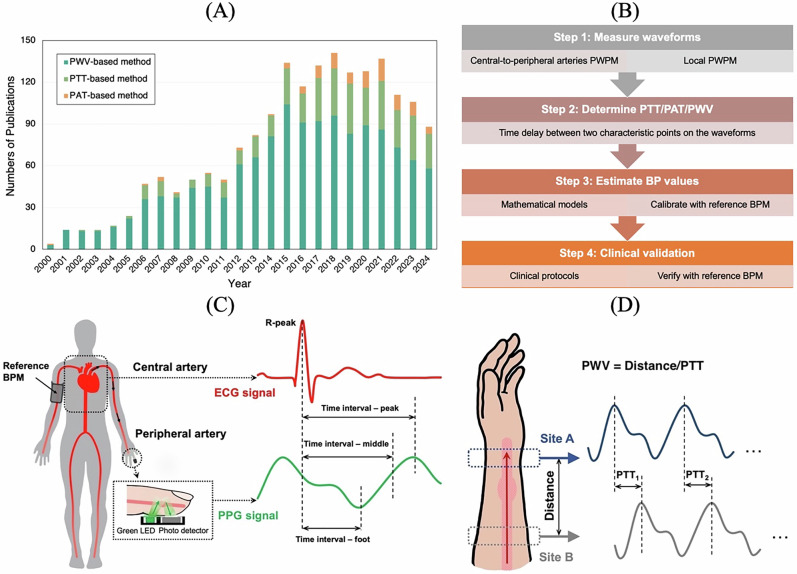
**A** The number of publications with the key words “PTT”, “PAT”, “PWV” and “BPM” (2000–2024). The data for the chart were extracted from www.scopus.com; **B** Four common steps of the development process of PWPM; **C** The most common ECG-PPG system for PAT determination; **D** The primary principle of the local-PWPM

Arterial stiffness and BP are closely interconnected in a bidirectional relationship [32]. Elevated BP imposes mechanical stress on the vascular walls, leading to elastic fiber degradation, endothelial dysfunction, and vascular smooth muscle contraction, all of which contribute to increased arterial stiffness. Since the early 2000s, researchers have been exploring and advancing a new cuffless BPM method, leveraging the well-established correlation between pulse wave propagation information and BP fluctuations. This is referred to as the Pulse Wave Propagation Method (PWPM) in this paper. Specifically, the time components of pulse wave propagation comprise pulse transit time (PTT), pulse arrival time (PAT) and pulse wave velocity (PWV). Research related to BP estimation using PWPM has witnessed a notable surge in scholarly attention over the past two decades, as evidenced by an analysis of publications retrieved from the Scopus Preview database, as depicted in Fig. 2A. Annual publication counts rose sharply from only 4 in 2000 to a peak of 141 in 2018. The PWV-based methodologies constitute the majority of research endeavors in this area, accounting for 73% of overall publications.

PWV is a gold standard for arterial stiffness assessment in clinical practice [33]. It can be defined as the velocity of a pulse wave propagating through two different arterial sites and can be calculated by a distance divided by the transit time.

1 $${PWV}=\frac{{Distance}}{{PTT}}$$

PTT was early determined by Weltman et al. in 1964 [34] by using the electrocardiogram (ECG) complex and a downstream pulse signal. PTT can be defined as the time taken for the blood pulse wave to travel from a proximal site to a distal site along the arterial tree. PAT is defined as the time interval between the R-peak of ECG signal and a characteristic point of the photoplethysmography (PPG) wave, which is equal to the sum of PTT and pre-ejection period (PEP). It is worth noting that PEP is not related to the blood pulse propagation; it consists of electromechanical delay and the time of isovolumic contraction [35]. However, some studies confound PTT and PAT, which might become misleading terminology.

First, a research group found the changes in PWV reliably followed the changes in BP through theoretical analysis and experiments on subjects [36]. Specifically, a linear relationship was found between PAT and MBP, and a non-linear decrease of PTT with increasing BP was measured in anaesthetized dogs [37]. The possibility and potential clinical application for humans were indicated in the following years [38, 39], including suitability for cardiopulmonary exercise conditions [40]. Figure 2B shows four common steps of the development process of PWPM. It should be emphasized that most cuffless BPM methods, including PWPM approaches, require calibration against a cuff-based reference device, and their ability to accurately track BP changes is fundamentally constrained by calibration stability and recalibration frequency.

Prior to this paper, several review articles have extensively summarized and analyzed research findings in the field of cuffless non-invasive continuous BPM [23, 41–45]. Researchers widely anticipate that cuffless BPM devices hold the potential to transform BPM, while overcoming significant challenges related to validation, accuracy, clinical integration is crucial to fully harness their capabilities and enhance hypertension awareness, treatment, and management. Although pulse wave analysis (PWA) has become increasingly popular and underpins several regulatory-approved cuffless BP devices, PWPM has a longer research history and a more direct physiological linkage to arterial stiffness and vascular properties. This review therefore focuses on PWPM to critically examine whether these methods remain promising for cuffless BP estimation or whether their limitations constrain future clinical translation.

Previous reviews have concentrated on either modeling or sensing technologies in isolation, with limited attention to clinical validation protocols. Furthermore, few studies have employed statistical frameworks to compare existing methods comprehensively. During the literature review process, we noted that researchers commonly reported the mean error within $\pm$ mmHg and standard deviation (SD) within $\pm$ mmHg to claim accuracy, often referencing outdated criteria from earlier standards such as ANSI/AAMI SP10:1992. However, this simplified validation neglected critical factors such as sample size (participant number), BP range, and demographic distribution, all of which are essential for valid performance evaluation according to ISO 81060-2:2019 [8]. These standards specify that the acceptable limits for error should not be static, but rather vary depending on the mean error and SD, ensuring that at least 85% of measurements fall within an acceptable error margin (*p̂* = 0.85). Given that many studies in this field, especially those at early research stages, did not meet the minimum sample size (*N* ≥ 85) or full BP range requirements due to practical constraints, relying on the $\pm$5/8 mmHg rule can lead to misleading conclusions.

In this review, the work is structured in two complementary parts. First, we provide an updated narrative review of advancements and challenges in BPM using PWPM, covering underlying physiological principles, mathematical models, and sensing technologies. Second, we perform a comparative re-evaluation of published studies using an enhanced statistical framework based on the Credence of Device Acceptability (CDA) and Probability of Tolerable Error (PTE) [46]. While many of these studies reported validation outcomes using heterogeneous protocols, this framework aligned with the statistical principles of ISO 81060-2:2019 and enables comparison by explicitly accounting for differences in sample size, BP range, mean error, and SD. This review provides a more rigorous and transparent assessment of the current performance and limitations of PWPM-based BPM systems, offering insights to guide future methodological development and clinically relevant evaluation.

## Methodology

### Review process

The literature search was designed to address two complementary research objectives:

1. to provide a narrative review of sensing techniques, mathematical models, and physiological assumptions underlying BPM based on PWPM; and
2. to identify studies that reported validation results for PWPM-based devices, enabling a comparative statistical re-evaluation using the CDA and PTE.

A structured narrative literature search was conducted up to June 2025, with primary focus on studies published within the past decade. Scopus and PubMed were used as structured bibliographic databases, while Google Scholar was employed as a supplementary source to capture gray literature and conference proceedings. Search terms included “non-invasive blood pressure measurement”, “continuous blood pressure measurement”, “cuffless blood pressure measurement”, “pulse wave velocity”, “pulse transit time”, and “pulse arrival time”. Studies were initially screened for relevance to PWPM-based cuffless BP estimation. Articles describing PWA approaches without explicit pulse propagation metrics were excluded but are briefly discussed where relevant for contextual comparison. Eligible studies were subsequently assessed for inclusion in the CDA and PTE analysis based on the availability of clinical validation data.

### Re-evaluation method

In accordance with international standards ISO 81060-2:2019, the measurement error for BP was defined as the difference between the reference BP and the corresponding reading from the device under test (DUT). A tolerable error threshold (typically $\pm$10 mmHg) was adopted to represent the maximum error considered clinically acceptable. Standards further specify a minimum confidence level (0.95) that a device is acceptable, defined as the probability that the true proportion of tolerable error exceeds a specified threshold (0.78), given an appropriate sample size and BP distribution.

To enable comparison across validation studies with differing sample sizes, BP ranges, and experimental designs, we applied the CDA framework, as previously described in [46]. CDA represents the probability that the proportion of tolerable errors observed for a DUT is no less than a reference threshold, which corresponds to the expected performance of a standards-compliant device if subjected to the same experimental design. This approach explicitly accounts for deviations from standardized validation conditions, including reduced sample size and non-uniform BP distributions. For each study, paired BP measurements and corresponding errors were modeled using a regression-based conditional error distribution. This was combined with the BP distribution stipulated by the relevant standard to obtain a joint probability density function of BP and error. The PTE was then calculated as the probability that measurement errors fall within tolerable error across the BP range. CDA was subsequently derived as the probability that the estimated proportion of tolerable errors exceeds the specified threshold. Together, CDA and PTE provide complementary metrics that reflect both per-measurement accuracy and overall confidence in device acceptability, offering a more informative assessment than conventional mean- and SD-based criteria alone.

## Techniques to determine pulse wave propagation

### Central-to-peripheral arteries methods

#### ECG-PPG system

The ECG-PPG method is the most typical system in the clinical setting to detect pulse wave propagation by calculating the time delay between a proximal ECG waveform and a distal PPG waveform (e.g., finger, wrist, ear, and toe), as shown in Fig. 2C. The time interval here is PAT, containing irrelevant PEP besides the requisite PTT. It was found a significant interindividual variability in PEP, particularly under mental and physical stress [47]. PAT should not be used as a marker of purely vascular function [48]. Another recent study comparing the invasive BP readings has demonstrated that PAT itself alone is not appropriate for BP estimation [49].

With the development of smart wireless devices, researchers attempted to extend the ECG-PPG system to wearable sensors for portable monitoring during daily activities, such as the computer mouse [50], smart vest [51], ear-worn device [52], smart watch [53], chest belt and wrist band in Body Sensor Network (BSN) systems [54–56]. ViSi Mobile System (SoteraWireless®, Inc, US) is a commercially available continuous monitoring for multiple healthcare parameters [57] which has been cleared for marketing by the Food and Drug Administration (FDA) [58].

#### Substitutions for ECG and PPG

The following techniques provide more optional measurements and have potential advantages over the ECG-PPG system.

- Ballistocardiogram (BCG) was used as a proximal timing reference in deriving PTT by detecting the reaction forces of the whole body to the cardiac ejection of blood into the aorta [59–61]. Recently, a research group combined the sensor into a wristband for limb BCG measurement [62, 63].
- Phonocardiogram (PCG) was also applied as the proximal timing reference [64, 65], which could detect two dominant types of sounds corresponding to the closure of mitral and tricuspid valves, and the closure of aortic and pulmonary valves.
- Seismocardiogram (SCG) is a non-invasive measurement to capture cardiac vibrations transmitted due to the heart’s mechanical activities from the chest wall by using accelerometers or gyroscopes [66]. It was recently applied to replace ECG as a surrogate proximal reference [67]. This group applied a gyroscope to estimate and eliminate the interferential PEP [68]. Their recent custom watch (SeismoWatch) could take multiple ambulatory BPM over 24 hours, paving another promising way toward long-term hemodynamic monitoring using PWPM [69].
- The two-PPG system is one feasible approach to detect PEP-free PTT, which was also able to be determined by time delay from the earlobe to fingers [70], from fingertips to the forehead temple [71], from shoulder to fingertips [72], from the left hand to the right hand [73], or from the palmar to dorsal sides of the wrist [74]. A commercial wrist watch (Biobeat BB-613WP, Biobeat Technologies Ltd, Israel) can track changes in BP based on PTT, which has been indicated as accurate by two validation studies [75, 76].
- Remote PPG (rPPG) signals were extracted from conventional RGB video recordings for a video-based non-contact BPM method [77]. A computer vision-based processing pipeline is employed to automatically detect and track facial and hand landmarks throughout the recording.
- Impedance plethysmography (IPG) is a typical application of bio-impedance measurement (BIM) for hemodynamic monitoring by detecting the volume change of the electrically conductive blood [78]. BIM can be applied as a proximal reference (at the subclavian artery), and then PTT determined from the impedance signal to the PPG signal at the radial artery [79]. BIM could also be placed at the wrist as the distal reference, and then PTT indicated by the time interval from the R-peak of ECG to the peak of the bio-impedance signal [80, 81].
- Flexible sensors, such as textile triboelectric nanogenerators, are emerging as compelling biotechnology for wearable pulse wave monitoring [82]. It is believed that flexible materials could crucially support the realization of wearable cuffless BPM, providing more comfort to wear and more cardiovascular health assessment as well as more electronics functionalities [83].
- Frequency-modulated continuous-wave (FMCW) radars were investigated for non-contact BP estimation by [84]. They measured the PTT between the chest and wrist by detecting skin displacement caused by the blood pulse using two radars. Although the accuracy of the estimation cannot be on par with contact technologies, their initial results pointed towards a promising future for BPM without any physical contact, ensuring safe and convenient measurements in unobtrusive and even invisible environments. A single-FMCW millimeter-wave radar was employed to extract reflective PTT (RPTT) from the wrist using a convolutional neural network (CNN) [85]. Unlike conventional PTT, RPTT is defined as the time delay between two peaks within the same cycle of a double-peaked pulse waveform.

### Local pulse wave propagation methods

Measuring local PWV is an emerging method for determining a host of cardiovascular events, such as arterial hemodynamic and local vessel pathologies [86]. The local-PWPM offers a distinct advantage by requiring minimal sensor placement area and specificity of the local artery’s distance, as depicted in Fig. 2 (D). Several techniques have been integrated into wearable wristbands for local-PWPM: BCG-PPG system [62], BIM-PPG systems [87, 88], two pressure sensor system [89], two PPG system [90], two BIM systems [91–95] and two PPG-ultrasound system [96]. PPG and BIM stand out as two prevalent techniques because of their high portability and low power consumption [92, 97]. Compared with PPG, BIA can detect volumetric and conductive changes in deeper arteries rather than superficial small vessels with less affected by skin pigmentation or ambient light. It also provides richer hemodynamic information related to arterial diameter and blood volume changes across different tissue layers [98].

## Mathematical models: the relationship between blood pressure and pulse wave propagation

According to the components of formulas, the mathematical models were classified into three categories: pulse wave propagation-only models (see Table 1), augmented components models (see Table 2) and ML-based models. Almost all listed models contained subjects’ coefficients which require initial calibration using reference BPM (RBPM).

**Table 1:** Pulse wave propagation-only models

Algorithm	Model number	Formula	Coefficients	Measured index	Reference
Proportional model	Model 1	${BP}=A\cdot {PTT}+B$ ${BP}=A\cdot {PAT}+B$	$A$ and $B$: subjects’ coefficients	PTT/PAT	[34, 38, 48, 60, 62, 72, 91, 97, 99, 105–109]
		${BP}=A\cdot {PWV}+B$		PWV	[79, 91, 110, 111]
Inverse model	Model 2	${BP}=\frac{A}{{PTT}}+B$		PTT	[64, 69, 73, 100, 112–114]
Inverse squared model	Model 3	${BP}=\frac{\Delta {BP}}{0.7}=\frac{\frac{1}{2}\rho \frac{{d}^{2}}{{{PTT}}^{2}}+\rho {gh}}{0.7}=\frac{A}{{{PTT}}^{2}}+B$	$d$: the blood flow distance, $g$: gravitational acceleration, $h$: the height between two measured sites, $\rho$: blood density, $A={(0.6\times {height})}^{2}\times \frac{\rho }{1.4}$, $B$: subjects’ coefficient	PTT	[85, 101, 113]
	Model 4	${BP}=a+{(\frac{b}{{PTT}-c})}^{2}$	$a$, $b$ and $c$: subjects’ coefficients	PTT	[40]
Logarithmic model	Model 5	${BP}=A\cdot {ln}\left({PTT}\right)+B$	$A$ and $B$: subjects’ coefficients	PTT	[59, 70, 77, 103, 113]
Exponent model	Model 6	$p=b{e}^{-\frac{k}{{PWV}}}$	$b$, $k$: subjects’ coefficients depend on age and gender	PWV	[104]

**Table 2:** Augmented components models

Augmented components	Model number	Formula	Coefficients	Measured index	Reference
Subject features	Model 7	${BP}=A\cdot {PTT}+B+C\cdot {HR}$	$A$, $B$ and $C$: subjects’ coefficients	PTT, HR	[115, 116]
	Model 8	${BP}={\beta }_{1}{{ln}}\left(\frac{{Hf}}{{PTT}}\right)+{\beta }_{2}$	$H$: height of subjects, $f$= 0.5, body correlation factor for adults to detect their peripheral pulse wave by fingers, ${\beta }_{1}$ and ${\beta }_{2}$: subjects’ coefficients	PTT/PAT	[65]
	Model 9	${BP}=a\cdot {Age}+b\cdot {BMI}+c\cdot {PWV}+d\cdot {HR}+e\cdot {MFCC}(1)+f\cdot {MFCC}(2)+g\cdot {MFCC}(6)+h\cdot {MFCC}(8)$	${BMI}$: body mass index, *MFCC* $({{\mathrm{1,2,6,8}}})$: mel-frequency cepstral coefficients, $a,b,c,d,e,f,g,h$: subjects’ coefficients	PWV, HR, speech sound	[117]
PPG features	Model 10	${BP}\left(i\right)={b}_{0}+{b}_{1}{PPG}\left(i\right)+{b}_{2}{PAT}\left(i\right)+{b}_{3}{PPG}\left(i-1\right)+{b}_{4}{PAT}\left(i-1\right)$	$i$: indicated beat-by-beat samples, ${PPG}$: the foot to peak amplitude of rising edge of the PPG wave, ${b}_{0}$, ${b}_{1}$, ${b}_{2}$, ${b}_{3}$ and ${b}_{4}$ : subjects’ coefficients	PAT, PPG amplitude	[118]
	Model 11	${DBP}={DB}{P}_{0}\cdot \frac{{PI}{R}_{0}}{{PIR}}$ ${SBP}={DB}{P}_{0}\cdot \frac{{PI}{R}_{0}}{{PIR}}+P{P}_{0}\cdot {(\frac{{PT}{T}_{0}}{{PTT}})}^{2}$ ${PIR}=\frac{{I}_{H}}{{I}_{L}}={e}^{\alpha \Delta d}$	${I}_{H}$: peak intensity of PPG signal, ${I}_{L}$: valley intensity of PPG signal, $\alpha$: a constant related to the optical absorption coefficients in the light path, The parameters with “0”: the initial calibration values by reference BP sphygmomanometer, $\Delta d$: diameter changes of the artery	PTT, PIR, Calibrated BP	[120, 121]
Basal conditions	Model 12	${P}_{e}={P}_{b}+\Delta P={P}_{b}-\frac{2}{\gamma \cdot {{PAT}}_{b}}\cdot \Delta {PAT}$	${P}_{b}$: the base SBP level, ${{PAT}}_{b}$: the time delay corresponding to ${P}_{b}$ *γ*: range from 0.016 to 0.018/mmHg	PTT, Base BP	[122]
	Model 13	${{BP}}_{{ptt}}=P1\times {PWV}\times {e}^{P3\times {PWV}}+P2\times {{PWV}}^{P4}-({{BP}}_{{ptt},{cal}}-{{BP}}_{{cal}})$	P1 to P4: subjects’ coefficients, ${{BP}}_{{ptt},{cal}}$: the calculated BP from PTT corresponding to the BP measured by the reference method, ${{BP}}_{{cal}}$: initial calibrated BP using the reference BPM.	PWV, Calibrated BP	[123, 132]
	Model 14	${DBP}=\frac{{{SBP}}_{o}}{3}+\frac{{2{DBP}}_{o}}{3}+A{{ln}}\left(\frac{{{PTT}}_{o}}{{PTT}}\right)-\frac{{{SBP}}_{o}-{{DBP}}_{o}}{3}\frac{{{PTT}}_{o}^{2}}{{{PTT}}^{2}}$ ${SBP}={DBP}+({{SBP}}_{o}-{{DBP}}_{o})\frac{{{PTT}}_{o}^{2}}{{{PTT}}^{2}}$	$A$: subjects’ coefficient, parameters with “o”: initial calibration with reference BP measurements,	PTT	[7, 124, 125], [133]
PTT variability and variation	Model 15	${BP}=\frac{A}{{PTT}}+B+C\cdot {VPTT}+D\cdot \left({PTTV}-{PTT}{V}_{0}\right)$ ${PTTV}=\sqrt{\frac{{\sum }_{i=1}^{N}{\left(\Delta {PT}{T}_{i}-{mean}\left(\Delta {PTT}\right)\right)}^{2}}{N-1}}$ ${VPTT}=\frac{{PTT}-{PT}{T}_{0}}{{PT}{T}_{0}}$	$A$, $B$, $C$ and *D*: subjects’ coefficients $\Delta {PTT}$: the difference between any two continuous PTTs, The parameters with “0”: the initially calibrated values in sitting postures, $N$: the number of PTT used for variability calculation ${PTTV}$: PTT variability during the signal recording, *VPTT*: the PTT variation at the measurement time.	PTT, VPTT, PTTV	[114]
Blood volume change	Model 16	${{SBP}}_{i}={k}_{s}{({C}_{{dx}})}_{i}^{2}+{{{\rm{a}}}}$ ${{DBP}}_{i}={k}_{d}{({C}_{{dx}})}_{i}^{2}+{k}_{{IHR}}{{IHR}}_{i}+b$	${IHR}$: the instantaneous heart rate, $i$: the number of pulses, ${k}_{s}$, ${k}_{d}$, ${k}_{{IHR}}$: fixed constants, ${{{\rm{a}}}}$ and $b$: subjects’ coefficients ${C}_{{dx}}$: related to both PWV and blood volume changes	PWV, Wave shape, HR	[51, 126]
	Model 17	${P}_{D}=\frac{2\rho {C}_{D}^{2}}{\beta }$ ${P}_{S}={P}_{D}{e}^{\beta \left(\frac{\Delta D}{{D}_{D}}\right)}$ $\beta =\frac{{ln}(\frac{P}{{P}_{D}})}{\left(\frac{D-{D}_{D}}{{D}_{D}}\right)}$	${P}_{D}$: end-diastolsic pressure corresponding to the diameter ${D}_{D}$, ${P}_{S}$: systolic-peak pressure, $P$: arterial pressure corresponding to lumen diameter $D$, $\rho$: density of blood, ${C}_{D}$: local PWV measured from the diastolic point, $\beta$: stiffness index $\Delta D$: distension of arterial diameter	PWV, arterial diameters	[96]
	Model 18	$P\left(t\right)={P}_{D}{e}^{\beta \left(\frac{D(t)}{{D}_{D}}-1\right)}$ $\beta =\frac{{ln}(\frac{{C}_{s}^{2}{D}_{D}}{{C}_{D}^{2}{D}_{s}})}{\left(\frac{{D}_{s}-{D}_{D}}{{D}_{D}}\right)}$	$\beta$: stiffness index ${D}_{D}$: arterial diameter at diastolic end ${D}_{s}$: arterial diameter at systolic end ${C}_{D}$: local PWV at diastolic end ${C}_{s}$: local PWV diameter at systolic end ${P}_{D}$: DBP	PWVs, arterial diameters	[127]
	Model 19	$p\left(t\right)={p}_{0}+\rho \frac{{D}^{2}}{{{PTT}}^{2}}{ln}\left[1+K({Z}_{0}-Z(t))\right]$	${p}_{0}$: base BP corresponding to ${Z}_{0}$, $\rho$: density of blood, $D$:distance between two sites, $K$: constant, ${Z}_{0}:$ impedance of the body segment, $Z(t)$: measured impedance	PTT, impedance	[87]
	Model 20	${DBP}={MBP}-k\cdot {PP}$ ${SBP}={MBP}-(1-k)$∙${PP}$ ${MBP}={ln}\left(\frac{{{PWV}}^{2}\cdot \rho \cdot D}{h\cdot {E}_{0}}\right)\cdot {\gamma }^{-1}{PP}=\frac{2\rho \cdot {{PWV}}^{2}\cdot {D}_{d}}{\beta D}{PIR}=\frac{{I}_{H}}{{I}_{L}}={e}^{-\alpha \cdot \pi \cdot D({D}_{s}-{D}_{d})}$	$k$: constant 0.76, $\rho$: density of blood, $D$: arterial diameter, $h$: wall thickness, *E*_*0*_$:$ elastic modulus at zero pressure, $\gamma$*:* coefficient 0.031, ${D}_{d}$: minimum diameter at diastole phase, ${D}_{s}$: maximum diameter at systole phase, ${I}_{H}$: peak intensity of PPG signal, ${I}_{L}$: valley intensity of PPG signal, $\alpha$: constant related to the optical absorption coefficients in the light path.	PWV, PIR	[129]
Pulse wave analysis	Model 21	${BP}={k}_{a}+{k}_{b}\cdot {sPWV}+{k}_{c}\cdot {sPWA}$	${k}_{a}$, ${k}_{b}$ and ${k}_{c}$: subject-specific multi regression model parameters, ${sPWV}:$ selective PWV from six single local-PWV, ${sPWA}:$ selective time-based pulse wave analysis parameters, including systolic peak, diastolic peak, dicrotic notch, second derivative maximum point, and valley point or systolic notch.	PWV and other time-based features	[130]
Adjusted PTT	Model 22	${BP}=A\cdot {APTT}+$ $B$ ${APTT}=\frac{{{PTT}}^{2}}{{RR}}$	$A,B$: subjects’ coefficients, ${APTT}$: adjusted PTT, ${RR}$: R-peak to R-peak interval of ECG waveform.	PTT and RR interval	[131]

### Pulse wave propagation-only models

- **Proportional model**

The most straightforward algorithm is the proportional model (Model 1), which obtained subjects’ parameters from linear regression analysis between measured PWV/PTT/PAT and corresponding reference BP. The repeatability of this model is questionable and a new calibration procedure was required after six months for a reliable estimation [99].

- **Inverse model**
  The inverse model (Model 2) is the simplest expression of the reciprocal relationship between PTT and BP. The wider application range of the inverse model was verified during increasing stress exercise, which indicated its potentiality for clinical applications [100].
- **Inverse squared model**
  The basic inverse squared model (Model 3) was proposed by expressing the pulse wave as the sum of the kinetic energy of the wave and the gravitational potential energy [101]. However, this model did not perform better than the inverse model in a recent evaluation study [102]. Another empirical non-linear model (Model 4) was proposed and compared to the simple proportional model based on the same set of data [40], demonstrating its more effective estimation than the proportional model.
- **Logarithmic model**
  Based on Bramwell-Hills and Moens-Kortweg’s formula, a logarithmic relationship can be obtained, and if the physical properties of the artery were constant for one subject, the simplified relationship between BP and PTT could be expressed as Model 5 [103].
- **Exponential model**
  An ordinary differential equation (Model 6) based on fundamental physics and physiology expressing the relationship between BP, Young’s modulus and PWV was proposed [104]. In this model, the age-/gender-dependent factors were combined in coefficients *b* and *k*. They used a benchmark model to calibrate the *b* and *k* for different age and gender groups.

### Augmented components

- **Subject features**
  The HR is a commonly augmented factor correlated with BP (Model 7) [105, 106]. The height and a body correlation factor were combined with the PWV-based logarithmic model [65]. As they reported, this model (Model 8) was robust enough to consistently estimate both SBP and DBP within grade A of the BHS protocol. In a recent study, Mel-frequency cepstral coefficients (MFCC) extracted from the /a/ vowel sounds detected in speech recordings were included alongside age and body mass index (BMI) variables (Model 9), both known to impact BP [107].
- **PPG features**
  A multiple linear regression combined with the foot to peak amplitude of rising edge of the PPG waveform (Model 10) was proposed by Chua and Heneghan [108]. The performance of estimated BP showed better results than using PAT-only model. A novel indicator named the photoplethysmogram intensity ratio (PIR) was proposed to evaluate the arterial diameter change [109]. Subsequently, this research group suggested DBP was related to PIR, and SBP corresponded to the PTT (Model 11). Their estimated SBP and DBP showed higher accuracy than other studies [110]. Another article applied the multi-adaptive regression spline (MARS) method based on the PIR model to achieve longer-time reliable BP estimations [111]. They indicated that the effective parameters (PTT and PIR) could represent the strong effects of the last state of the cardiopulmonary system on BP values.
- **Basal conditions**
  Model 12 was proposed by simplifying the well-known Bramwell-Hills and Moens-Kortweg’s formula in 2000 [112]. This research intermittently calibrated the estimation using the low-frequency component (basal SBP) measured by a RBPM, while the accuracy highly relied on the re-calibration interval. In order to reduce the inconvenience of interval calibration, Model 13 was presented as a combination function consisting of an exponential term, a second non-linear term, and a one-point correction constant [113].
  Based on the assumption that the MBP is approximately the sum of a third of SBP and two-thirds of DBP, Poon and Zhang [7] estimated SBP and DBP based on the logarithmic relationship between MBP and PTT with an initial calibration of SBP and DBP (Model 14). Some studies also specified $A=\frac{2}{\gamma }$ [114, 115]. It showed no consistent correlations between estimated BP and the RBPM during the daytime while obtaining a better negative correlation during night time [115]. They explained two main factors that influenced the relationship between PTT and BP: 1) the vascular tone caused by daytime activities, and 2) the physiological status such as stress and emotion. Lately, the impact of heart disease on the accuracy of Model 14 was investigated by Ding et al. [114], observing a more accurate DBP estimation in patients.
- **PTT variability**
  A study indexed the variation and variability of PTT (the definition of VPTT and PTTV was shown in Table 2) as the indication of neural control and integrated this with the original inverse model to obtain Model 15, providing a better estimation, especially for DBP [116]. This improvement demonstrated neural control as one crucial BP regulation mechanism and should be considered in future research.
- **Blood volume and arterial diameter**
  As early in 2000, Heard et al. [117] achieved accurate estimation in DBP and MBP by using Model 16 and ECG-PPG system “DxTek monitor” (DxTek, Inc., Chestnut Hill, MA). Besides adding HR component, the parameter ${C}_{{dx}}$ was related to both PAT and $\frac{\triangle V}{V}$. Arterial diameter changes serve as a surrogate for blood volume variations during the cardiac cycle. An ultrasound-PPG probe was developed for real-time BP evaluation [96]. Arterial diameter-related components were directly captured via ultrasound (Model 17). A pair of piezoelectric micromachined ultrasound transducers (PMUTs) with flexible encapsulation were employed to simultaneously measure local PWVs and arterial diameter waveforms at both systolic and diastolic ends to indicate arterial stiffness index, enabling calibration-free BPM (Model 18) [118].
  For a specific segment, if PWV was assumed as a relative constant within a cardiac cycle, the pressure can be expressed in terms of local PWV and the cross-sectional area [119]. The cross-sectional area was presented using the variation of magnitude impedance, as shown in Model 19 [87]. In a recent advancement, the PIR has been enhanced and integrated into Model 20 model for calibration-free BP estimation [120]. The device incorporated a pair of piezoelectric ceramic pressure sensors to detect radial artery PWV. Additionally, ultrasound imaging was utilized to measure the radial artery’s diameter while simultaneously recording the PIR signal for modeling purposes.
- **Hybrid selective PWV and PWA**
  By incorporating nine additional time-based features from PWA—such as systolic and diastolic peaks, dicrotic notch, and derivative points—the hybrid PWV–PWA model (Model 21) achieved higher accuracy than PWV alone, offering a promising approach to enhance PWPM reliability [121].
- **Adjusted PTT**
  The adjusted pulse transit time (APTT) was explored for monitoring beat-to-beat femoral SBP during ventricular arrhythmia in patients undergoing radiofrequency ablation [122]. APTT, defined as the ratio of squared conventional PTT to the ECG RR interval, reflects arterial elasticity and stroke volume (Model 22).

### Models based on machine learning

Recent advances in artificial intelligence (AI) have greatly expanded the use of machine learning (ML) for BP estimation, enabling automated data learning and prediction [123]. Early studies showed that artificial neural networks (ANNs) outperformed traditional regression methods for SBP estimation [124]. Subsequent work using Support Vector Regression (SVR) and additional features from the PPG’s second derivative improved accuracy by up to 40% [125]. Other approaches, such as AdaBoost regression for BIM-based PWPM [94] and hybrid models like “genetic algorithm-mean impact value-support vector regression” (GA-MIV-SVR) [126], further enhanced performance. Feature optimization using genetic algorithms and double-layer multilayer perceptron ANN also achieved promising results [90]. Deep convolutional neural networks achieved a high accuracy of BP estimation (86.3%) without explicit feature extraction [127]. A comparison study evaluated the effectiveness of multiple ML algorithms and revealed that RF models achieved the highest predictive performance, reaching an overall accuracy of approximately 90% in classifying BP categories based on PTT-derived features [128]. A multimodal approach combining PAT, pulse wave morphology, and demographic data with advanced ML algorithms—including Lasso, Random Forest (RF), Support Vector Machine (SVM), Artificial Neural Network (ANN), and Long Short-Term Memory (LSTM) was proposed [129]. The results underscored the importance of integrating both temporal and morphological features for accurate BP estimation. Similarly, accurate BP estimation was achieved by incorporating morphological and computational features from symmetric PPG signals into an attention-based CNN–biLSTM framework [130]. As the focus of this paper is not on ML, readers who want to understand more details about ML-based modeling, are referred to other recent review articles for continuous non-invasive BPM [131–133].

## Re-evaluation of previous clinical studies

### Summary of previous studies

Researchers commonly adopt established protocols such as ANSI/AAMI/ISO, BHS, and ESH. However, for early-stage studies, conducting a full validation strictly following these standards can be challenging, resulting in findings that may be difficult to compare or justify. In this review, we summarized the clinical validation protocols reported in previous studies. A total of 47 studies published since 2000 were initially identified. Those published before 2015 or lacking sufficient information (e.g., fewer than 10 participants, unspecified BP ranges, or missing mean error and SD) were excluded. Ultimately, 22 studies (24 datasets) conducted between 2015 and 2025 were included, and their original validation details are organized in Table 3, followed by a re-evaluation using the CDA and PTE metrics. In this table, several articles did not describe clearly the specific model of reference sphygmomanometers, which thus were filled with the general word “cuff-based”. In this table, numbers linked by “-” presented the range of parameters, and numbers lined by “$\pm$” presented the means $\pm$ standard deviation (SD) of the data, BP range marked with “*” was the approximate value estimated from the charts/figures in articles.

**Table 3:** Clinical protocols of studies (*2015 to 2025*, *N: Sample size; M: Male; F: Female; RE*: *Relative error; MAD (mmHg): mean absolute difference/mean absolute errors/mean estimation errors between estimated BP values and reference BP values; SD (mmHg): standard deviation; Mean *${{{\boldsymbol{\pm }}}}\,$*SD (mmHg): mean difference/error* $\pm$ standard deviation for estimated BP values against reference BP values; **RMSEs** (mmHg): Root-mean-squared-errors between estimated BP and reference values; **R**: correlation coefficients between estimated BP values and reference BP values; r^*^: correlation coefficients between changes of PAT/PTT and changes of reference BP values; **r**: correlation coefficients between measured parameters (e.g., PWV, PAT, PTT, etc.) and reference BP values; *r*^2^: correlation coefficients between the PAT/PTT and reference BP values; R^2^ (Mean $\pm \,$SD): coefficients of determinations between PTT and reference BP values.)

Studies	Participant information							Measurement method				Reported Accuracy	Re-evaluation Results			
	*N*	Sex	Age	Healthy conditions	Measured condition	BP Range		Techniques	Measured index	Model	RBPM		SBP		DBP	
						SBP	DBP						CDA	PTE	CDA	PTE
[58]	30	M	20–83	Healthy and hypertension	Meet the requirement of ISO 81060-2:2013			*ViSi Mobile System (ECG-PPG)*	PAT	ML	Oscillometry (GE DINAMAPTM CARESCAPETM V100)	Mean $\pm \,$SD = -4.20$\pm$8.70 (SBP), -3.80$\pm$5.80 (DBP)	0.01	0.65	0.07	0.78
	49	F														
[120]	14	M	25.6$\pm$2.1	healthy, non-smoker	Seated position	115.19$\pm$4.87	73.25$\pm$2.98	ECG-PPG	PTT, PIR, base BP	11	Finapres	Mean $\pm \,$SD = -0.37$\pm$5.21 (SBP), -0.08$\pm$4.06 (DBP) MAD = 4.09 (SBP), 3.18 (DBP)	0.26	0.84	0.8	0.93
	13	F														
[124]	21	M	27.4$\pm$ 10	healthy	Seated at rest (three trails: first trail, 2 weeks, one month after)	105.65$\pm$10.70	62.50$\pm$7.50	ECG-PPG	PTT, initial BP	14	Auscultation	Mean $\pm \,$SD = 0.50$\pm$11.55 (SBP), 1.19$\pm$9.07 (DBP)	0.01	0.56	0.01	0.67
	16	F														
	16	M	80.7$\pm$ 12	patients		134.03$\pm$17.28	63.00$\pm$9.55					Mean $\pm \,$SD = -3.6$\pm$16.02 (SBP), -1.06$\pm$7.92 (DBP)	0.01	0.43	0.03	0.76
	32	F														
[64]	24	M, F	21–50	healthy	Sit upright after with 3-min running at 8 km/h	106–197	51–93	PCG-PPG	PTT	2	Oscillometry (Model M3, OMRON, Japan)	MAD = 7.47 (SBP), 3.56 (DBP) R = 0.84 (SBP), 0.86 (DBP) Mean $\pm \,$SD = 0.00$\pm$11.08 (SBP), 0.00$\pm$4.53 (DBP)	0.01	0.61	0.93	0.96
[112]	24	M	21–50	healthy	Sit upright after with 3-min running at 8 km/h; Train group: 17 Test group: 15	106–197	51–93	PCG-PPG; ECG-PPG	PTT; PAT	2	Oscillometry (Model M3, OMRON, Japan), mercury	PCG-PPG (PTT) method: Mean $\pm \,$SD = -0.28$\pm$9.44 (SBP), 1.03$\pm$5.15 (DBP) MAD = 6.22 (SBP), 3.97 (DBP) R = 0.89 (SBP), 0.84 (DBP) ECG-PPG (PAT) method: Mean $\pm \,$SD = 0.12$\pm$6.15 (SBP), 1.31$\pm$5.36 (DBP) MAD = 4.71 (SBP), 4.44 (DBP) R = 0.95 (SBP), 0.84 (DBP)	0.01	0.69	0.83	0.93
	8	F											0.53	0.88	0.73	0.91
[91]	9	M	30$\pm$5	healthy	Seated with the arm resting on a table; handgrip exercise	95–165*	50–110*	BIM	PWV	1	Oscillometry (Oscar 2, SunTech Medical®, US)	Mean $\pm \,$SD = 0.01$\pm$8.10 (SBP), -0.06$\pm$5.46 (DBP) R = 0.81$\pm$0.08 (SBP), 0.84$\pm$0.07 (DBP) RMSEs = 7.48$\pm$2.15 (SBP), 5.17$\pm$1.81 (DBP)	0.04	0.75	0.6	0.92
	6	F														
[133]	6	M	37$\pm$9	four hypertensives	Calibration: every 2 days for 2 weeks; Validation: 1 time per week for 4 weeks	136$\pm$14	84$\pm$12	ECG-PPG	PTT, HR, respiratory rate	14	Auscultation	Mean $\pm \,$SD = 1.04$\pm$6.88 (SBP), -2.16$\pm$6.60 (DBP)	0.09	0.78	0.1	0.79
	4	F														
[96]	63	M	57$\pm$12 (24–85)	Healthy (40), Hypertensive (43)	Seated at rest after 3–5 min relax	144$\pm$19	85$\pm$12	Ultrasound-PPG	PWV, artery dimensions	17	Oscillometry (SunTech 247, SunTech Medical, North Carolina, US)	DBP: R = 0.86 RMSEs = 8.30 Mean $\pm \,$SD = 3.80$\pm$1.96	NA		0.98	0.92
	20	F														
[145]	21	M	25.6$\pm$2.1 (21–31)	healthy	Non-smokers without caffeine ingestion within 6 h, sitting quietly	108–132*	77–86*	ECG-PPG	PTT, RR interval	ML	Finapres	R = 0.90$\pm$0.07 (SBP), 0.79$\pm$0.10 (DBP) Mean $\pm \,$SD = -0.36$\pm$2.37 (SBP), 1.07$\pm$3.23 (DBP) MAD = 1.95 (SBP), 2.15 (DBP)	0.99	0.99	0.99	0.97
	21	F														
[90]	100	M	32.63$\pm$15.82	healthy	Without any specific medicine	85–185*	60–110*	Two-PPG	PWV, PTT and PPG features	ML	Oscillometry (Model M6 Comfort, OMRON, Japan)	MAD = 4.94 (SBP), 4.03 (DBP) Mean $\pm \,$SD = 0.00$\pm$5.90 (SBP) 0.53$\pm$5.50 (DBP) R = 0.94 (SBP), 0.84 (DBP)	0.98	0.91	0.99	0.92
	11	F														
[87]	10	M	29$\pm$5	healthy	Relax, handgrip exercise, and recovery in the seated position	85–160*	55–115*	IPG-PPG	PTT, radial impedance	19	Oscillometry (Oscar 2, SunTech Medical®, US)	Mean $\pm \,$SD = 0.31$\pm$8.55 (SBP), -0.5$\pm$5.07 (DBP) R = 0.88$\pm$0.07 (SBP), 0.88$\pm$0.06 (DBP) RMSEs = 8.47$\pm$0.91 (SBP), 5.02$\pm$0.73 (DBP)	0.02	0.72	0.7	0.94
	5	F														
[63]	17	M	23$\pm$5	healthy	Resting: standstill Changes: cold pressor; mental arithmetic; slow breathing; breath-holding.	70–190*	40–120*	Wrist BCG-PPG	PTT and 6 BCG features	ML	Finapres	R = 0.77$\pm$0.04 (SBP), 0.80$\pm$0.04 (DBP) RMSE = 9.10$\pm$1.10 (SBP), 6.00$\pm$0.70 (DBP) MAD = 7.20$\pm$0.90 (SBP), 4.70$\pm$0.50 (DBP) Mean $\pm \,$SD = 0.00$\pm$9.30 (SBP), 0.00$\pm$6.20 (DBP)	0.01	0.7	0.46	0.88
	6	F														
[69]	16	M	25.9$\pm$3.4	healthy	12 measurements over 24 h except sleeping	80–140*	50–100*	SCG-PPG	PTT	2	Oscillometry	RMSE = 4.75 (SBP), 2.72 (DBP) MAD = 4.03 (SBP), 2.24 (DBP) Mean $\pm \,$SD = 0.00$\pm$5.44 (SBP), 0.00$\pm$2.88 (DBP)	0.58	0.91	0.99	0.99
	5	F														
[117]	17	M	19-49	healthy	Rest with intermittent vocalization	107–153	55–93	ECG-PPG, Speech recording	PWV, age, BMI, HR, MFCC	9	Oscillometry (Model M3, OMRON, Japan)	R = 0.61 (SBP), 0.60 (DBP) MAD = 8.06 (SBP), 7.48 (DBP) Mean $\pm \,$SD = 0.01$\pm$9.69 (SBP), 0.04$\pm$9.68 (DBP)	0.01	0.66	0.01	0.67
	5	F	19-23			104–148	53–99									
[129]	89	M	<30 (14%) 31–40 (76.7) >60 (9.3%)	healthy	Rest condition 60 s with three replications	125.5$\pm$13.0	80.7$\pm$8.7	piezoelectric ceramics as pressure sensors and PPG	PWV, PIR	20	Auscultation	R = 0.97 (SBP), 0.90 (DBP) Mean $\pm \,$SD = 2.1$\pm$ 3.4 (SBP), 0.8$\pm$4.2 (DBP)	0.99	0.97	0.99	0.97
	40	F														
[141]	23	-	-	healthy	Rest: 5 min	100–160*	50–100*	Two-PPG	6 PTT features and 24 PPG features	ML	Oscillometry	R = 0.92 (SBP), 0.88 (DBP) Mean $\pm \,$SD = 1.65$\pm$1.91 (SBP), 2.16$\pm$2.03 (DBP)	0.99	0.99	0.99	0.99
[146]	13	M	20–30	healthy	Rest: sit in the chair with a relaxed posture for 30 s, repeated 5 times	90–170*	45–95*	ECG-PPG	PAT and other PPG features	ML	Oscillometry (OMROM HEM-7211)	Mean $\pm \,$SD = -0.3$\pm$3.63 (SBP), -0.3$\pm$2.75 (DBP) MAD = 5.21$\pm$5.98 (SBP), 4.15$\pm$5.66 (DBP)	0.97	0.98	0.99	0.99
	2	F														
[131]	27	M	41.4$\pm$14.3	patients with ventricular arrhythmia (VA), frequent premature ventricular contraction (PVC) or supraventricular tachycardia (VT)	Underwent radiofrequency ablation for arrhythmias at Hospital	132.8$\pm$23.8 (VA) 129.7$\pm$24.2 (PVC) 134.4$\pm$21.8 (VT)	77.1$\pm$13.9 (VA) 74.3$\pm$18 (PVC) 81$\pm$20 (VT)	ECG-PPG	PTT and RR interval	22	Invasive method	SBP: RMSE = 8.22$\pm$3.39 Mean $\pm \,$SD = -0.01$\pm$10.54 R = 0.95	0.01	0.64	NA	
	18	F														
[147]	11	M	23.6$\pm$1.6	healthy	Rest, Physical exercise	126.3$\pm$14.6	73.3$\pm$10.2	ECG-BIM (brain)	42 features (e.g., PTT, PAT, HR and signals morphology)	ML	Oscillometry (BSX-533, Haier, China)	Mean $\pm \,$SD = 0.39$\pm$4.55 (SBP), -0.07$\pm$3.55 (DBP) MAD = 2.17 (SBP), 1.71 (DBP) R = 0.9 (SBP), 0.89 (DBP) RMSE = 3.91 (SBP), 3.02 (DBP)	0.77	0.95	0.95	0.98
	2	F														
[74]	13	M	24.8$\pm$3.1	healthy	1 min measurement with 10 to 20 times	90–130*	55–80*	Two-PPG at wrist	8 PPG features, 4 interface sensor features and 6 subject characteristics	ML	Oscillometry (OMROM J751)	Mean $\pm \,$SD = 0.44$\pm$6.00 (SBP), -0.50$\pm$6.20 (DBP)	0.19	0.83	0.14	0.82
	5	F														
[85]	10	M	26–55	healthy	Sit on the chair and place his/her wrist on the desk (palms facing up), remaining stationary for 60 s	90–150	60–100	single-frequency-modulated continuous-wave millimeter-wave radar	Reflective PTT	3 and ML	HKG-08B Sensor	Mean $\pm \,$SD = -1.3$\pm$6.17 (SBP), -3.1$\pm$4.93 (DBP)	0.23	0.84	0.23	0.84
	5	F														
[127]	12	M	22–36	healthy	Seated in a relaxed position	90.75–130.05	64.26–84.83	Two-ultrasound	PWVs, arterial diameter	18	Oscillometry (YE680CR, Yuwell)	Mean $\pm \,$SD = 2.64$\pm$1.82 (SBP) Mean $\pm \,$SD = 1.76$\pm$1.31 (DBP)	0.89	0.97	0.99	0.99
	5	F														

In this paper, studies were compared and re-evaluated based on ISO 81060-2 rather than ISO 81060-3 because ISO 81060-3 was only issued in 2022, and to date we have not identified any published studies that have incorporated ISO 81060-3 requirements into their clinical protocols. ISO 81060-2 stipulates a prerequisite of a minimum of 85 participants from the general population and at least 35 individuals from special populations (45 for pregnancy), applicable across all patient demographics. However, among the studies examined in Table 3, only 2 studies involved a sufficient number of subjects. Moreover, merely 5 studies targeted unhealthy subjects, particularly those afflicted with cardiac diseases. A larger sample size holds the potential to enhance statistical power and accuracy, enabling more thorough subgroup investigations over the long term. The commonest device to obtain reference BP values was the oscillometry devices (approximately 68.18%). Standards suggested that the auscultation with two professional observers shall be performed as the critical reference. Different protocols have been designed to elevate participants’ BP such as running, bicycle ergometer, handgrip, stair climbing and walking, thereby covering a wider BP range—baseline, exercise and recovery. There are ten statistical analysis methods (parameters) presented in previous studies from three views: (1) Bias: the difference between their results (estimated BP values) and their targets (reference BP values); (2) Variation/dispersion: standard deviations of the difference between various methods; (3) Correlation: the linear relationship between estimation values and target values, which was a common approach to select more relevant features.

### Overview of re-evaluation

This paper referenced statistical criteria consistent with ISO 81060-2, which specify a minimum sample size of *N* $\ge$85 and appropriate BP distributions (i.e., SBP: 130 $\pm$ 20 mmHg; DBP: 80 $\pm$ 13 mmHg). In our previous research [46], we developed a novel statistical evaluation framework, which explicitly incorporates sample size, BP distribution, mean error and SD into the analysis. The framework defines the CDA, representing the probability that a DUT meets acceptable performance criteria under given experimental conditions. Moreover, through statistical modeling of error distributions, regression analyses, and the joint density of BP and measurement error, it estimates the likelihood that a DUT would satisfy international standards even when study conditions deviate from the ideal requirements. The online calculator powered by Wolfram Cloud was used for the re-evaluation [134]. When studies reported only the BP range (minimum and maximum), the mean and SD of BP were estimated using the method proposed by [135].

The CDA does not have a direct regulatory equivalent and is intended as an interpretative and comparative metric rather than a binary pass-fail criterion. CDA quantifies the probability that a device would satisfy standardised accuracy requirements when accounting for experimental uncertainties and variations. Its interpretation depends on the study objective. For early-stage or feasibility studies with limited sample sizes, a CDA $\ge$0.5 suggests that the device is more likely than not to demonstrate acceptable accuracy in subsequent larger scale validation, indicating promising performance. For confirmatory validation studies, higher CDA values are desirable, reflecting greater confidence in true device accuracy. In this review, accuracy assessment was therefore interpreted in the context of probability-based acceptability, with particular emphasis on high-confidence performance (e.g., acceptance probabilities $\ge$95%), in line with the confidence principles underpinning existing standards.

The PTE is grounded in existing international standards. In ANSI/AAMI SP10:2002 (Annex F), a tolerable error of $\pm$10 mmHg is defined, and a device is considered acceptable if the estimated probability that its measurement error lies within this range is at least 85%. Consistent with this rationale, a PTE $\ge$0.85 was therefore adopted as the threshold in the present analysis.

The reported accuracies and re-evaluated results have been listed in Table 3. CDA can reflect the level of confidence that the proposed BPM method would pass the ISO 81060-2 if repeated under ideal, fully compliant conditions. PTE can indicate the likelihood that a randomly selected individual’s BP would be measured within a clinically acceptable error margin (e.g., $\pm$10 mmHg). Across the studies, a positive relationship between CDA and the PTE can be found. Studies with stronger methodological rigor and validation credibility are more likely to achieve acceptable accuracy in real-world conditions. However, a wide inter-study variability remains evident, especially at CDA values lower than 0.4, where the PTE range from approximately 0.55 to 0.85. This dispersion suggests that device acceptability alone does not fully guarantee consistent population-level performance, emphasizing the importance of comprehensive validation of new BPM, including subject demographics and physiological variability.

According to the SP10 standard, BPM devices are required to maintain a mean error within $\pm$ mmHg and a SD not exceeding 8 mmHg. Based on the originally reported data, 13 datasets satisfied these criteria for SBP, while 20 datasets met the requirements for DBP. However, our re-evaluation yielded a more stringent assessment of performance: only five studies achieved CDA values exceeding 0.95 for both SBP and DBP. Overall, DBP estimation demonstrated superior accuracy compared with SBP. Specifically, among studies that also achieved a promising PTE ($>$0.85), 14 studies attained CDA values above 0.5 for DBP, whereas only 8 studies met this criterion for SBP. It is imperative to acknowledge that even studies falling below these thresholds can offer valuable insights for the advancement of BPM. Despite the relatively lower evaluation outcomes observed in these studies, it is essential to emphasize that this critique does not diminish their potential contributions to the field.

### Studies with high CDA and PTE

For SBP results, there are five studies achieved CDA values over 0.95 with PTE over 0.85. Among these studies, three studies focused on measuring local pulse wave propagation and two studies utilized the conventional ECG-PPG system. Notably, all studies incorporated additional features or information beyond PAT/PTT/PWV, such as arterial diameter-related index, RR-interval, and PIR index. Moreover, signal morphology-based features have emerged as valuable tools for improving BP estimation accuracy. By analyzing the shape and characteristics of physiological signals (e.g., PPG), researchers can extract additional information related to cardiovascular function. Among the nine studies that employed ML, four achieved a high CDA over 0.95. Notably, these four studies accounted for the majority of the high performing studies, as four of the five studies with CDA $>$0.95 incorporated ML. This suggests that while ML is not sufficient on its own to guarantee reliable BP estimation, it may play an important contributory role when combined with appropriate physiological features.

The accuracy of DBP estimation appears less variable across studies compared with SBP, likely due to the narrower physiological range of DBP values. Specifically, 9 out of 22 studies achieved relatively high CDA scores above 0.95. The algorithms employed ranged from conventional regression-based models to advanced ML frameworks. Similar to the SBP results, almost all studies incorporated additional physiological or morphological indices rather than relying solely on pulse wave propagation.

### Limitations of the present re-evaluation

It should be acknowledged that ISO 81060-2:2019 was originally developed for intermittent, cuff-based sphygmomanometers and does not explicitly address the defining characteristics of cuffless BPM, particularly continuous BP and the ability to track BP changes over time. ISO 81060-3:2022 has been developed for continuous automated BP type, while ISO/CD 81060-7 is under development to address intermittent or repeated-intermittent cuffless type. In parallel, IEEE 1708a-2019 and the ESH recommendations place particular emphasis on BP trend tracking following calibration and on evaluating device performance under dynamic physiological conditions. Together, these frameworks highlight that validation strategies should be selected according to the intended measurement modality and clinical application, and that researchers should follow the guideline most appropriate to the operating principle and use case of their device.

In the present review, ISO 81060-2 was therefore not used as an endorsement of its suitability for cuffless BPM, but rather as a common reference framework to enable systematic comparison across prior PWPM studies that predominantly relied on ISO-style accuracy metrics. Importantly, our re-evaluation highlights a key limitation of the existing literature: among the 22 studies included, only six applied BP perturbation protocols (e.g., exercise, handgrip, or postural changes), and only one study explicitly assessed BP trend-tracking performance. The majority of studies evaluated accuracy under relatively static conditions, often immediately following calibration. Because CDA and PTE values were calculated based on the reported paired measurements within each study, high CDA and PTE do not necessarily translate to reliable BP estimation after BP changes without recalibration. Therefore, we suggested future validation should also consider BP change tracking and calibration stability, in addition to static accuracy assessments.

## Suggestions for future work

### Techniques

Based on the re-evaluation results, no single technical approach demonstrated a clear advantage over others. The sensing techniques appear to function primarily as tools for acquiring signals from two distinct sites—what ultimately matters is their ability to provide accurate timing and high-fidelity waveform information for pulse wave propagation analysis. Therefore, future development should place greater emphasis on form factor and contextual applicability. The design should consider how the device will be used in its intended setting—whether in a controlled clinical environment as ECG-PPG systems already provided, as an affordable home-based monitoring system, or as part of a continuous wearable platform—balancing measurement precision with user comfort, convenience, and long-term reliability.

The ECG-PPG system stands out as a typical method, offering the ability to discern the time delay between proximal-distal signals with relatively better affordability and ease of integration. Nevertheless, isolating or compensating for PEP effects remain key methodological challenges. PPG sensors provide valuable insights into blood volume variations and vascular compliance. A latest meta-analysis demonstrated that the PPG-based PWA approach slightly outperformed conventional PWPM, although the difference was not statistically significant [136]. Similar to PPG sensors, BIM has emerged as a promising technique worthy of further exploration for extracting arterial diameter-related information, potentially enabling a more direct link between arterial diameter changes and BP [98, 137].

Despite attempts at substitution, proposed systems have struggled with significant signal noise stemming from motion artifacts and changes in position - particularly the contactless technologies (e.g., camera or radar sensors). Considering real-life scenarios, the suppression of motion artifacts and environmental noise remains a significant barrier to the reliable implementation of continuous BPM. Future advancements in measurement techniques should prioritize the development of robust noise-cancellation strategies or intelligent systems capable of detecting and isolating stable measurement conditions in dynamic environments.

### Mathematical models

The overall relationship between pulse wave propagation and BP has been well established, allowing BP values to be estimated using a single pulse propagation index. However, the calibrated coefficients may vary with the subject’s physiological state. Consequently, an initially calibrated model may lose accuracy when the subject’s BP falls outside a certain range or changes over time. To address these limitations, researchers have implemented various compensation strategies to improve model reliability. Overall, the accuracy of BP estimation critically depends on both the quantity and quality of the BP-related indices extracted and utilized in the mathematical model. The pulse waveform can provide more information beyond serving as a time reference, and has also been widely investigated in what is known as the PWA method:

1. Amplitude information: PPG amplitude as a sympathetic marker was considered a component of BP estimation [108]. Analogously, PIR was also used to evaluate the modulation of sympathetic nervous activity on BP [110].
2. Horizontal information: time-scale parameters can be extracted from the pulse waveform, such as cardiac period, systolic upstroke time and diastolic time [138, 139], time interval between an early systolic peak and a later diastolic peak of the PPG signal [140], the descent time from diastolic peak to the end of diastole [141], the rise time from the nadir to the systolic peak of the PPG waveform associated with left ventricular function [142], and the ratio of participant’s height to the time difference between the systolic peak and the diastolic peak as the artery stiffness index [143].

The integration of diverse features derived from waveform morphology has become a prevalent strategy across various ML-based models, facilitating the augmentation of BP-related information accessible for estimation purposes. Such parameters normally offset the low-frequency influences, which cannot be tracked by pulse wave propagation. The rhythmic oscillations of BP can be identified with the appearance in its spectrum as individual peaks, which reflect: 1) High frequency: the oscillation frequency is normally between 0.2 and 0.35 Hz, which is similar to that of respiratory activity; 2) Low frequency: oscillations with a frequency approximately from 0.1 to 0.15 Hz are associated with vasomotion waves caused by an oscillation of the sympathetic vasomotor tone. The mechanisms of the fluctuation of BP with respiration can be explained as the intrathoracic pressure change with breathing which has a mechanical impact on venous return, pulmonary vascular, and aortic pressure, therefore, causing the oscillations of BP [144]. Early research evidenced the low-frequency component of BP variation caused by local changes in smooth muscle constriction and dilation through the modulation of the sympathetic nervous activity [145]. The small variation in peripheral arterial diameter caused by smooth muscle represents a large change in the arterial cross-sectional area and produces a large impact on the resistance of blood flow [146]. Therefore, the diameter change of the artery is one component that can reflect the low-frequency effects on BP. In the past decade, the arterial diameter variation was directly measured using ultrasound [96, 118] and indirectly expressed as magnitude impedance [87] or PPG intensity ratio [110]. A recent study [147] with 20 participants found that carotid PWV increased with BP in only 4 individuals who showed large BP fluctuations. No significant BP-PWV correlation was seen in others with smaller variations, suggesting PWPM may be less sensitive to minor BP changes. Recent work [148] further emphasizes the need to consider temporal PWV variations, as using a constant PWV across the cardiac cycle can cause major BP prediction errors.

Therefore, future work is expected to extract more useful BP-related information from measured waveforms, including factors reflecting neural control, sympathetic activity, and arterial diameter changes. ML-based approaches may offer a more efficient means of estimating BP by facilitating the selection of relevant features. However, based on our review and evaluation, the use of ML models does not inherently guarantee reliable BP estimation. While some studies have reported high CDA and a high PTE, a larger number of studies have not achieved such accuracy.

### Calibration process

We have similar conclusion that although extensive research and technological advancements have been devoted to PWPM, these methods still cannot offer accuracy improvements beyond what could be achieved by traditional devices [149]. According to the present research progress and products in the market (e.g., *ViSi Mobile System* [57]*, Biobeat wristwatch* [150]*, and Aktiia 24/7 BPM* [151]), it may be difficult to avoid the calibration procedure. According to the latest ISO 81060-3 standard for continuous methods, calibration and recalibration procedures (initialization and re-initialization) have been incorporated into the clinical validation process, as they directly influence both the study protocol design and the classification of the device’s intended use. User tolerance for calibration and recalibration may be a key factor determining the potential market adoption of PWPM-based BP devices. If the process is perceived as cumbersome, users may be less willing to maintain long-term usage, which in turn can affect the device’s commercial success. We believe AI can play a pivotal role in this process by learning complex patterns from large datasets to enable personalized BP estimation. Establishing a comprehensive database encompassing diverse populations - across different ages, genders, and health conditions - could further minimize the need for individual calibration and enhance model generalizability.

### Clinical validation and standards

The regulatory framework for cuffless BP devices has struggled to keep pace with the rapid advancements in technology and their direct accessibility to consumers. The standardized testing protocols has garnered widespread recognition and has been underscored in numerous review articles [23, 29, 30, 45, 133, 149, 152–154]. We strongly advise caution in using cuffless BP devices, especially in clinical settings or even among healthy individuals, until more robust accuracy data is available.

Our re-evaluation results evident that the conventional criteria of “mean error $\le$5 mmHg and SD $\le$8 mmHg” is insufficient on its own to reliably demonstrate methodological accuracy, even for static validation. Within the specific context of ISO 81060-2 based static accuracy assessment, we recommend that early-stage researchers supplement these criteria with the CDA and PTE metrics to better quantify agreement strength and population-level performance. Although the ISO 81060-2 standard was used as the reference protocol in this review, its intrinsic purpose focused on intermittent devices rather than tacking BP changes, with CDA and PTE serving as supportive but not substitutive accuracy metrics. Thereby, for studies targeting continuous or repeated intermittent BPM, particularly those involving BP variability or trend tracking, evaluation should follow standards specifically developed for those applications rather than ISO 81060-2:

1. ISO 81060-3:2022 [20] is the first standard specifically addressing continuous non-invasive sphygmomanometers, defined as devices that estimate BP from each cardiac cycle and provide a continual series of BP parameters with an output period of $\le$30 s. It introduces rigorous requirements, including independent measurement framework, repeated-pair requirements, intended-use categorization (Type A for absolute BP and Type T for trending BP), change evaluation interval, worst case output settings, and etc. It also incorporates assessments of device stability (robustness of performance over time), and the capability to reliably track dynamic BP changes. However, it mandates the use of invasive intra-arterial BP as the reference, which might be technically and ethically challenging for early investigations and small-scale pilot studies.
2. IEEE 1708 Standard for Wearable, Cuffless Blood Pressure Measuring Devices [19] followed by an amendment in 2019 (IEEE 1708a-2019) addresses methodological challenges unique to cuffless BP measurement, which evaluates both continuous and intermittent cuffless devices using manual auscultatory BP as the reference and incorporates validation across multiple conditions: static measurements immediately following calibration, assessments during induced BP increases and decreases (up to 30 mmHg), and evaluations conducted prior to recommended recalibration.
3. The ESH guidance [21] focuses primarily on intermittent cuffless devices, defined as systems providing BP estimates at intervals longer than 30 s, which currently represent the most prevalent category of cuffless BPMs. To accommodate the heterogeneity of these devices, the ESH framework first establishes standardized terminology covering BP output frequency (continuous or intermittent), measurement mode, sensing modality, and calibration strategy, and subsequently categorizes cuffless BPMs into nine device types. Based on this classification, six complementary validation tests are proposed to assess different aspects of device performance: a static test for absolute BP accuracy; a device position test to evaluate robustness against hydrostatic pressure effects; treatment and exercise tests to assess accuracy during BP decreases and increases, respectively; an awake/asleep test to examine BP change tracking across physiological states; and a recalibration test to assess calibration stability over time. The required validation pathway is tailored according to whether the device relies on individual calibration, performs automatic or user-initiated measurements, and operates across multiple body positions.

## Supplementary information

Supplementary information

Supplementary information

Supplementary information
