# Supplementary material for: Advancements and challenges in blood pressure monitoring using pulse wave propagation: a comprehensive review and ISO 81060-2 based statistical analysis
Source: Hypertens Res. 2026 May 7;49(7):2156–80. doi: 10.1038/s41440-026-02651-3 (PMC13333497; doi:10.1038/s41440-026-02651-3)
Supplement: Supplementary file 1 — Supplementary information [file 41440_2026_2651_MOESM1_ESM.docx]

# Mathematical Models

PWV is a gold standard for arterial stiffness assessment in clinical practice . It can be defined as the velocity of a pulse wave propagating through two different arterial sites and can be calculated by a distance divided by the transit time.

|  | $PWV=\frac{Distance}{PTT}$ | Equation 1 |
| --- | --- | --- |

As early as 1922, the PWV (in cm/s) was used as an index of distensibility, giving a basic relationship with BP change ($\Delta P$ in dyne/cm^2^) and blood volume change ($\Delta V$)/ cross-sectional area change ($\Delta A$):

|  | $PWV=\sqrt{\frac{V\Delta P}{\rho\Delta V}}=\sqrt{\frac{A\Delta P}{\rho\Delta A}}$ | Equation 2 |
| --- | --- | --- |

where $V$ is the initial volume, $A$ is the initial cross-sectional area, and $\rho$ is the density of blood. Expressing $\Delta P$ in mm/Hg and PWV in m/s, then substituting $\rho$=1.055, the above equation becomes:

|  | $PWV=0.357\sqrt{\frac{V\Delta P}{\Delta V}}=0.357\sqrt{\frac{A\Delta P}{\Delta A}}$ | Equation 3 |
| --- | --- | --- |

Later in 1963, PWV was also expressed by the elasticity of arteries in the well-known Moens-Korteweg formula by assuming the artery as an elastic tube:

|  | $PWV=\sqrt{\frac{Eh}{\rho d}}$ | Equation 4 |
| --- | --- | --- |

where *h* is the thickness, *E* is Young's modulus of the arterial wall, *d* is the inner arterial diameter, and $\rho$is the density of blood. Arterial walls will expand during systole and contract during diastole with blood flow passing by, and the dynamic elastic modulus of central arteries increases exponentially with increasing BP:

|  | $E=E_{0}e^{\gamma P}$ | Equation 5 |
| --- | --- | --- |

where $E_{0}$ is the elastic modulus at zero pressure, *P* is the mean BP (mmHg), and $\gamma$ is the subject-dependent coefficient ranging from 0.016 to 0.018 (mmHg^-1^). Combining **Error! Reference source not found.**, Equation 4, and Equation 5, a relationship between BP and PWV/PTT is then given by:

|  | $PWV=\frac{Distance}{PTT}=\sqrt{\frac{hE_{0}e^{\gamma P}}{\rho d}}$ | Equation 6 |
| --- | --- | --- |
