# Supplementary material for: Advancements and challenges in blood pressure monitoring using pulse wave propagation: a comprehensive review and ISO 81060-2 based statistical analysis
Source: Hypertens Res. 2026 May 7;49(7):2156–80. doi: 10.1038/s41440-026-02651-3 (PMC13333497; doi:10.1038/s41440-026-02651-3)
Supplement: Supplementary file 2 — Supplementary information [file 41440_2026_2651_MOESM2_ESM.docx]

# Literature search strategy and study selection

The literature search was designed to address two complementary objectives:

1. to provide a narrative review of sensing techniques, mathematical models, and physiological assumptions underlying blood pressure monitoring (BPM) based on pulse wave propagation metrics (PWPM); and
2. to identify studies reporting validation results for PWPM-based cuffless BP devices, enabling comparative statistical re-evaluation using the Credence of Device Acceptability (CDA) and Probability of Tolerable Error (PTE).

A structured narrative literature search was conducted up to June 2025, with primary emphasis on studies published within the past decade. Scopus and PubMed were used as the primary bibliographic databases, while Google Scholar was employed as a supplementary source to capture grey literature and conference proceedings. Search terms included combinations of “non-invasive blood pressure measurement”, “continuous blood pressure measurement”, “cuffless blood pressure measurement”, “pulse wave velocity”, “pulse transit time”, and “pulse arrival time”. The complete search strategy and terms are provided in the Supplementary Material.

Studies were initially screened for relevance to PWPM-based cuffless BP estimation. Articles focusing exclusively on pulse wave analysis (PWA) without explicit pulse propagation metrics were excluded from quantitative analysis but are briefly discussed where relevant for contextual comparison. For the narrative review, a total of 168 references were included, comprising 153 journal and conference articles, 5 standards and guidelines, and 10 web-based or other resources.

For the validation-focused analysis, the review concentrated on clinical protocols and reported accuracy metrics. A total of 47 studies were selected for further detailed review and re-evaluation. Studies published before 2015, or those lacking sufficient validation information (e.g., fewer than 10 participants, unspecified BP ranges, or missing mean error and standard deviation), were excluded. Ultimately, 22 studies (24 datasets) published between 2015 and 2025 met the eligibility criteria and were included in the CDA and PTE analyses based on the availability of clinical validation data.

A flow chart summarizing the study identification, screening, inclusion, and exclusion process is provided in the Supplementary Material. As this work is not a conventional systematic review, the PRISMA flow diagram was adapted to reflect the narrative review structure and the additional quantitative re-evaluation component.
