# Supplementary figures and images for: Advancements and challenges in blood pressure monitoring using pulse wave propagation: a comprehensive review and ISO 81060-2 based statistical analysis
Source: Hypertens Res. 2026 May 7;49(7):2156–80. doi: 10.1038/s41440-026-02651-3 (PMC13333497; doi:10.1038/s41440-026-02651-3)

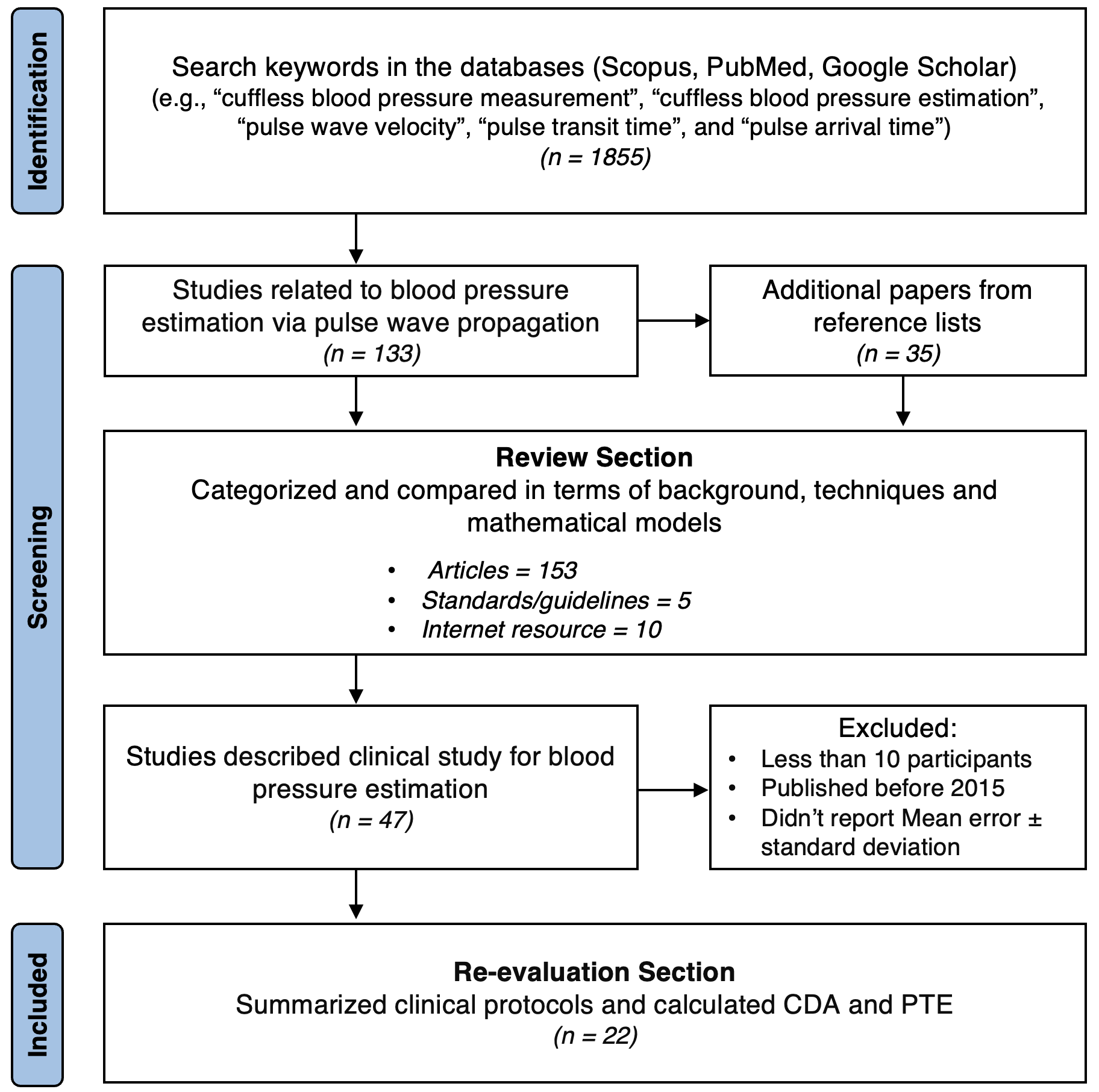

Supplement: Supplementary file 3 — Supplementary information [file 41440_2026_2651_MOESM3_ESM.png]
